# Association of Neighborhood Geographic Spatial Factors With Rates of Childhood Obesity

**DOI:** 10.1001/jamanetworkopen.2018.0954

**Published:** 2018-08-03

**Authors:** Di Fang, Michael R. Thomsen, Rodolfo M. Nayga, Anthony Goudie

**Affiliations:** 1 Department of Agricultural Economics and Agribusiness, University of Arkansas, Fayetteville; 2 Arkansas Center for Health Improvement, University of Arkansas for Medical Sciences, Little Rock

## Abstract

**Importance:**

Childhood obesity is a principal public health concern. Understanding the geographic distribution of childhood obesity can inform the design and delivery of interventions.

**Objective:**

To better understand the causes of spatial dependence in rates of childhood obesity across neighborhoods.

**Design, Setting, and Participants:**

This cohort study used data from a legislatively mandated body mass index screening program for public school children in Arkansas from the 2003-2004 through 2014-2015 academic years. Spatial autoregressive moving average (SARMA) models for panel data were used to estimate spatial dependency in childhood obesity at 2 levels of spatial aggregation. Data were analyzed from August 2017 to February 2018.

**Exposures:**

The SARMA models included geographic fixed effects to capture time-invariant differences in neighborhood characteristics along with controls for the mean age of children and the proportion of children by race/ethnicity, school meal status, and sex.

**Main Outcomes and Measures:**

The proportion of obese schoolchildren in Arkansas neighborhoods by year, defined at larger (census tract) and smaller (census block group) spatial scales.

**Results:**

The geographic aggregations were based on 935 800 children with a mean (SD) age of 132 (39) months. Of these children, 51% were male; 65% were white, 21% were black, 10% were Hispanic, 2% were Asian, and the remainder were of other or unidentified race/ethnicity. In models without geographic fixed effects, there was evidence of positive and significant spatial autocorrelation in obesity rates across tracts (ρ = 0.511; 95% CI, 0.469-0.553) and block groups (ρ = 0.569; 95% CI, 0.543-0.595). When geographic fixed effects were included, spatial autocorrelation diminished at the census tract level (ρ = 0.271; 95% CI, 0.147-0.396) and disappeared at the census block group level (ρ = −0.075; 95% CI, −0.264 to 0.114).

**Conclusions and Relevance:**

Because block groups are smaller than tracts, children in neighboring block groups were more likely to attend the same schools and interact through neighborhood play. Thus, geographic-based social networks were more likely to span block group boundaries. The lack of evidence of spatial autocorrelation in block group–level models suggests that social contagion may be less important than differences in neighborhood context across space. Caution should be used in interpreting significant spatial autocorrelation as evidence of social contagion in obesity.

## Key Points

**Question:**

Is social contagion associated with spatial patterns in childhood obesity rates across neighborhoods in Arkansas?

**Findings:**

In this cohort study of 935 800 children, after controlling for neighborhood fixed effects, positive and significant spatial autocorrelation was detected using spatial panel models when obesity rates were computed for larger census tracts but not when computed for smaller census block groups, indicating that neighborhood contextual factors, rather than social contagion, appeared to better explain observed spatial patterns in obesity rates across neighborhoods.

**Meaning:**

Spatial analysis may be used in epidemic studies, but researchers should use caution when interpreting a positive spatial autocorrelation as evidence for contagion, especially in a social context.

## Teaser

This study uses census block data in Arkansas to investigate spatial patterns in childhood obesity between the 2003-2004 and 2014-2015 academic years and to distinguish obesity risk attributable to social contagion vs neighborhood contextual factors.

## Introduction

Childhood obesity is a persistent problem in the United States, with 17% of children having obesity.^1^ A finding in the literature is that the incidence of obesity can cluster through social networks.^2,3^ Although obesity is not a disease that can be transmitted through contact, the analog remains that children who reside in close proximity are more likely to form friendships, which in turn can lead to the spread of obesity through the development of common habits or by altering one’s body type to identify with peers. This phenomenon could be defined broadly as social contagion and could be associated with spatial dependency similar to that found in studies of contagious diseases. According to Datar and Nicosia,^4^ social contagion means that any member of a social network who becomes obese affects the likelihood that others in the network will also become obese through social influences.

Social contagion, if present, would manifest in spillovers in obesity rates across geographic units or, more specifically, in positive spatial autocorrelation, because of children being likely to form neighborhood-based friendship networks. Geographic location is the primary factor in assignment of children to public schools, which are an important venue for the formation of childhood friendship networks. Spatial autocorrelation emerges in the presence of social contagion because social network relationships, although dependent on geography, do not necessarily respect arbitrary geographic boundaries. Support for this argument can be found in studies of infectious diseases, in which the mechanism of spread is through contact between infected individuals, and there is evidence of positive spatial autocorrelation in rates of infection across geographic units.^5,6,7,8,9,10,11,12,13^ There is also evidence of positive spatial autocorrelation in rates of childhood obesity^14,15^ and adult obesity^16,17^ across geographic units.

The existence of spatial autocorrelation, however, cannot be taken as prima facie evidence of social contagion. The problem is that obesity can show spatial clustering for reasons unrelated to socialization. Manski^18^ elucidates the problems associated with separating endogenous peer effects arising from a process such as social contagion from contextual effects and correlated effects. Contextual effects result from characteristics common to members of a social network, such as neighborhood safety, access to green space, and annual days with weather conducive to outside play. Correlated effects emerge from the tendency of a group to behave similarly not because of socialization but because they share similar personal characteristics or institutional environments.^18^ To the extent that children in nearby geographic areas are subject to similar environmental features or share common personal or familial characteristics that impact diet or physical activity, spatial autocorrelation arises if these features are dependent on space but are not adequately captured in the statistical model. In short, spatial autocorrelation could be evidence of social contagion, but as shown by McMillen,^19^ can also reflect an inadequate model specification.

In this study, we used information from a unique longitudinal data set resulting from a legislatively mandated body mass index (BMI) (measured as weight in kilograms divided by height in meters squared) screening program to investigate whether spatial autocorrelation could be better explained by social contagion or by shared environmental and personal characteristics. Although the data do not provide information on social networks, they permit us to examine obesity rates over time using 2 different levels of spatial aggregation that represent a division into larger (more aggregate) and smaller (less aggregate) geographic units. Because of the longitudinal nature of these data, geographic fixed effects could be used to account for time-invariant, unobservable neighborhood-level or community-level characteristics that may not have been adequately controlled in earlier cross-sectional studies. If social contagion better explains spatial autocorrelation, it should be most pronounced across the smaller geographic units because these reflect divisions over which childhood social networks are most likely to span. Moreover, spatial autocorrelation that is associated with social contagion should continue to be present even after inclusion of fixed effects to account for contextual, personal, and family characteristics that characterize the geographic unit.

## Methods

This study used data collected from a legislatively mandated BMI screening program of public school children in Arkansas (Act 1220 of 2003). Parents or children could opt out of this program. The use of these data for this research project was reviewed by the institutional review board at the University of Arkansas and was determined to meet exemption 4 for “research involving the collection or study of existing data or specimens if publicly available or information recorded such that subjects cannot be identified.” The University of Arkansas Institutional Review Board protocol number is 14-07-026. This study followed the Strengthening the Reporting of Observational Studies in Epidemiology (STROBE) reporting guideline. The childhood obesity rate in Arkansas ranks among the highest in the United States, and Arkansas became the first US state to institute a BMI screening program for public school children. All schoolchildren were measured annually from the 2003-2004 academic year through the 2006-2007 academic year. Afterward, children were measured biennially in even-numbered grades (kindergarten and grades 2, 4, 6, 8, and 10). Standard protocols and equipment are used to measure BMI in schools across the state. Data from this program are maintained by the Arkansas Center for Health Improvement (ACHI), and the ACHI compiles annual reports of the screening program by academic year.^20^ The ACHI facilitated development of the data set for this research.

### Participants and Measures

We examined obesity rates over a 12-year period (academic year 2003-2004 through academic year 2014-2015) across 2 levels of spatial aggregation: census tracts and census block groups. The census tracts are the larger of the 2 units and, according to the US Census Bureau, are designed to optimally contain approximately 4000 people.^21^ Census block groups are subdivisions of tracts and are the smallest unit at which we can feasibly characterize geography in this study. In the 2010 census geography, there are 686 tracts and 2147 block groups in Arkansas. The 2010 census geography was used for all 12 years of our sample.

We calculated the proportion of obese schoolchildren in each census block group and in each tract in Arkansas for each academic year. The obesity rate was defined as the proportion of children in the tract or block group with a BMI *z* score above the 95th percentile using the standard reference growth curves from the Centers for Disease Control and Prevention.^22^ We also computed the mean age in years of children by tract or block group along with proportions of children by race/ethnicity and school meal status. The school meal status measures reflect the proportion of low-income children in the tract or block group. Children from households with an income less than 130% of the poverty level qualify for free meals, and children from households with an income less than 185% of the poverty level qualify for reduced-price school meals.^23^ After these aggregations, we had two 12-year panel data sets: one for census tracts and another for census block groups. The methods described below require nonmissing values for each tract or block group. Depending on the year, between 1 and 3 of the 686 tracts and between 6 and 9 of the 2147 block groups had missing proportions. To avoid artificially inducing special dependence into these data, the missing values for these few block groups or tracts were replaced with the state averages.

### Statistical Analysis

The study used spatial autoregressive moving average (SARMA) models.^24,25^ Details of this model are provided in the eAppendix in the Supplement. The SARMA models may have an advantage because they are able to separate spatial dependency caused by social contagion from that caused by the contextual environment. This is done through the construction of weight matrices that place higher weights on neighboring geographic units.^24,25,26^

There are 2 considerations in the estimation of a SARMA model. First, a study area needs to be divided into nonoverlapping geographic units. A modifiable areal unit problem occurs when studies that use aggregate data do not distinguish between spatial associations created artificially by the aggregation and real associations presented by the individuals within the geographic units.^27^ Given this problem, the estimate of spatial effects could be sensitive to the manner of aggregation. Second, McMillen^19^ cautioned that autocorrelation is often produced spuriously by model misspecification, such as omitted variables, and he argued that the high degree of parameterization of spatial lag and spatial error models could induce an incorrect structure for the covariance.

A common remedy for the omitted variable issue raised by McMillen^19^ is the inclusion of spatial fixed effects.^28^ According to Anselin and Arribas-Bel,^28^ a spatial fixed effects specification is appropriate when individual observations are organized into well-delineated groups and some characteristics of the group are unobserved. For example, when physical education classes vary by school district but no data are available to measure the performance of the schools, a spatial fixed-effects variable can capture how this variation is reflected in the prevalence of obesity. Geographic fixed effects will reduce spatial autocorrelation resulting from inadequate specification of contextual factors. However, Anselin and Arribas-Bel^28^ stated that spatial fixed effects could address only a form of spatial heterogeneity and not true spatial dependence. They argued that, if true spatial dependence is present, spatial fixed effects would not remove this dependence, with the only exception being that the spatial autocorrelation takes on a group-wise structure that is the same as the spatial unit. In terms of our study, the implication is that true spatial dependence, such as that associated with social contagion, should still be detectible even if fixed effects successfully account for dependency arising from contextual and correlated effects as described by Manski.^18^

As Manski^18^ notes, endogenous peer effects, contextual effects, and unobserved correlated effects can all contribute to similar behaviors and are inherently difficult to separate with linear-in-mean models. The panel SARMA model allows for such separation because the endogenous effect is calculated as the association of the weighted average obesity rate of neighboring units (block group or tract) with the obesity rate of the focal unit. Assuming the unobserved effect also follows a spatial pattern, the correlated effect is addressed in part by the geographic fixed effect and in part by applying the spatial weight matrix to the error term.

Given these considerations, we estimated SARMA models using both the tract and block group panels described above. If spatial autocorrelation is associated with social contagion, it should be more pronounced across the smaller census block groups. This is because neighborhood-based friendship networks are more likely to extend across block group boundaries than across tract boundaries. Moreover, children in neighboring block groups are more likely to be in the same public school catchment areas and therefore more likely to be in the same school-based social networks than children in neighboring tracts.

Using similar reasoning, compared with tract effects, block group effects would better capture characteristics of the microenvironment that could explain geographic differences in obesity rates because children in the same block group are more likely to attend the same schools, have similar school nutrition and physical activity programs, and be homogeneous with respect to access to parks and safe places for vigorous play. Thus, the strongest evidence of social contagion would be indicated by spatial autocorrelation in the block group panel after inclusion of block group effects.

The SARMA models were estimated using the spml package in R, version 3.4.3 (R Foundation).^29^ The feature of primary interest is the spatial autoregressive term (ρ) obtained from the different model specifications. The models also included a spatial error term (λ), which accounts for unobserved correlated effects not captured in the geographic fixed effects or in other model covariates. A standard *z* score (normal distribution) was used to calculate *P* values, and 2-sided distribution was used. *P* < .05 was considered to be statistically significant. Data were analyzed from August 2017 to February 2018.

## Results

### Participants

The census tract and block group aggregations used in this study were based on 935 800 children (51% male) with a mean (SD) age of 132 (39) months. At least 1 valid weight status indicator was available for 689 809 of these children. Sixty-five percent of the children were white, 21% were black, 10% were Hispanic, 2% were Asian, and the remainder (2%) were of other or unidentified race or ethnicity.

### Descriptive Statistics

Summary statistics for the tract and block group aggregate data sets used in the SARMA models are presented in Table 1. The mean values were similar across the 2 samples. There were higher SDs in the block group panel because block groups are smaller geographic units and reflect less aggregation.

**Table 1.** Model Variables for Census Tract-Level and Block Group–Level Panels^a^

Variable	Census Tract-Level Panel (n = 8232)	Census Block Group–Level Panel (n = 25 746)
Obesity prevalence	0.22 (0.06)	0.22 (0.08)
Race/ethnicity		
Black	0.24 (0.29)	0.24 (0.31)
Asian	0.02 (0.03)	0.02 (0.04)
Hispanic	0.07 (0.11)	0.07 (0.12)
Other	0.01 (0.02)	0.01 (0.02)
Female sex	0.49 (0.03)	0.49 (0.05)
Eligible for free school meals^b^	0.50 (0.21)	0.51 (0.23)
Eligible for reduced-price meals^b^	0.10 (0.05)	0.10 (0.07)
Mean age, y	11.04 (0.35)	11.03 (0.48)

^a^ Unless otherwise specified, data are mean (SD) proportion of students within the census tract or census block group.

^b^ Eligibility for free and reduced-price school meals represents the proportion of children from lower-income families in the census tract or block group. Children from families with incomes less than 130% of the poverty level are eligible for free meals. Children from families with incomes between 130% and 185% of the poverty level are eligible for reduced-price meals.^23^

### Primary Analysis

Estimates from the tract-level and block group–level spatial models are reported in Table 2. Table 2 presents estimates from a non–spatial panel model as a reference point along with SARMA models estimated with year fixed effects only and with both geographic fixed effects and year fixed effects. Using a conditional Lagrange multiplier (LM) test, we rejected the null hypothesis of a non–spatial panel model in favor of the SARMA model for both the tract and block group data sets. For the tract-level panel, the test statistic was LM = 6.189 (*P* < .001). For the block group–level panel, the test statistic was LM = 7.920 (*P* < .001).

**Table 2.** Coefficient Estimates for Census Tract-Level and Block Group–Level Models^a^

Variable	Non–Spatial Panel Data Model With Time and Geographic Fixed Effects	SARMA Model Without Geographic Fixed Effects	SARMA Model With Time and Geographic Fixed Effects
**Census tract-level models (n = 8232)**			
Spatial error term, λ	NA	−0.251 (−0.321 to −0.181)	−0.252 (−0.404 to −0.100)
Spatial autoregressive term, ρ	NA	0.511 (0.469 to 0.553)	0.271 (0.147 to 0.396)
Race/ethnicity			
Black	−0.060 (−0.086 to −0.034)	−0.011 (−0.015 to −0.007)	−0.058 (−0.081 to −0.034)
Hispanic	0.096 (0.058 to 0.134)	0.051 (0.043 to 0.06)	0.088 (0.055 to 0.122)
Asian	−0.074 (−0.162 to 0.013)	−0.214 (−0.253 to −0.175)	−0.062 (−0.141 to 0.018)
Other	−0.458 (−0.522 to −0.395)	−0.36 (−0.409 to −0.311)	−0.447 (−0.506 to −0.388)
Female sex	0.006 (−0.023 to 0.035)	−0.011 (−0.037 to 0.016)	0.004 (−0.023 to 0.031)
Eligible for free school meals^b^	0.027 (0.012 to 0.043)	0.089 (0.082 to 0.096)	0.024 (0.011 to 0.038)
Eligible for reduced-price school meals^b^	0.006 (−0.014 to 0.026)	0.096 (0.08 to 0.113)	0.003 (−0.013 to 0.020)
Mean age, y	0.003 (0.000 to 0.006)	0.003 (0.000 to 0.006)	0.003 (0.000 to 0.005)
Year effects	Yes	Yes	Yes
Tract effects	Yes	No	Yes
**Census block group–level models (n = 25 764)**			
Spatial error term, λ	NA	−0.456 (−0.5 to −0.412)	0.124 (−0.052 to 0.3)
Spatial lag term, ρ	NA	0.569 (0.543 to 0.595)	−0.075 (−0.264 to 0.114)
Race/ethnicity			
Black	−0.003 (−0.022 to 0.015)	−0.007 (−0.009 to −0.004)	−0.002 (−0.019 to 0.016)
Hispanic	0.087 (0.062 to 0.111)	0.032 (0.026 to 0.038)	0.087 (0.063 to 0.11)
Asian	0.137 (0.097 to 0.177)	−0.06 (−0.081 to −0.039)	0.137 (0.098 to 0.176)
Other	−0.208 (−0.271 to −0.145)	−0.307 (−0.349 to −0.265)	−0.204 (−0.264 to −0.143)
Female sex	−0.062 (−0.079 to −0.044)	−0.052 (−0.067 to −0.038)	−0.062 (−0.078 to −0.046)
Eligible for free school meals^b^	0.024 (0.012 to 0.035)	0.074 (0.07 to 0.079)	0.024 (0.012 to 0.035)
Eligible for reduced-price school meals^b^	0.008 (−0.007 to 0.023)	0.074 (0.063 to 0.085)	0.009 (−0.007 to 0.024)
Mean age, y	0.009 (0.007 to 0.011)	0.006 (0.005 to 0.008)	0.009 (0.008 to 0.011)
Year effects	Yes	Yes	Yes
Block group effects	Yes	No	Yes

Abbreviations: NA, not applicable; SARMA, spatial autoregressive moving average.

^a^ Unless otherwise specified, data are estimated proportion of students within the census tract or census block group (95% CI). The dependent variable is proportion of children with obesity in the census tract or census block group.

^b^ Eligibility for free and reduced-price school meals represents the proportion of children from lower-income families in the census tract or census block group. Children from families with incomes less than 130% of the poverty level are eligible for free meals. Children from families with incomes between 130% and 185% of the poverty level are eligible for reduced-price meals.^23^

As discussed above, our focal interest was spatial autocorrelation across the tracts or census block groups. This is captured by the estimate for the spatial autoregressive parameter (ρ). A comparison of ρ across the 2 SARMA models at the tract level revealed positive and significant spatial autocorrelation. The inclusion of tract fixed effects caused the estimate of spatial autocorrelation to decrease by nearly one-half, from ρ = 0.511 (95% CI, 0.469-0.553; *P* < .001) to ρ = 0.271 (95% CI, 0.147-0.396; *P* < .001). In contrast, a comparison of ρ across the 2 SARMA models at the block group level showed that there was no longer significant spatial autocorrelation after inclusion of block group effects. The inclusion of block group effects caused the estimate of spatial autocorrelation to decrease from ρ = 0.569 (95% CI, 0.543 to 0.595; *P* < .001) to ρ = −0.075 (95% CI, −0.264 to 0.114; *P* = .44).

Additional insight is provided by the spatial error term (λ). As noted above, λ accounts for spatial dependence in the model errors. The λ continued to be significant in the tract-level panel even after including fixed tract effects (λ = −0.252; 95% CI, −0.404 to −0.100; *P* = .001) (Table 2). By contrast, λ was no longer significant in the corresponding model with block group fixed effects (λ = 0.124; 95% CI, −0.052 to 0.3; *P* = .17). This indicates that the smaller, block group effects better captured unobserved contextual features that influenced obesity rates.

Covariate estimates in Table 2 provided additional insights into the importance of the tract and block group fixed effects in the model. A comparison of estimates from the non–spatial models with those from the SARMA models with tract and block group effects revealed similar point estimates. This was expected because the point estimates are unbiased regardless of spatial dependency in the model errors. Point estimates from the models without tract or block group fixed effects were similar in sign, but the point estimates diverged, suggesting that the geographic fixed effects were accounting for contextual and correlated factors that were not directly observed.

Although these findings were not the central focus of this study, estimates in Table 2 suggested that neighborhoods with a higher proportion of Hispanic children were associated with higher rates of obesity, and neighborhoods with a higher proportion of children qualifying for free school meals were also associated with higher rates of obesity. The finding of a negative association with obesity for neighborhoods with higher proportions of black children was unexpected, but this finding could be explained by regional differences across the state. Statewide, 222 of 2147 block groups and 64 of 686 census tracts were predominantly composed of black children (>80% of the children in these block groups and tracts were black), and these block groups and tracts tended to be concentrated in urban areas, such as Little Rock. There were differences across the tract and block models for estimated associations between the obesity rate and the proportion of Asian children and girls. This reflected the higher variation across block groups compared with tracts.

### Secondary Analyses

The choice of the weight matrix and the resulting specification of distance decay could lead to different spatial effects.^19^ In this study, the weight matrix captured the empirical path through which social contagion was associated with obesity rate. The weight matrix used in the models reported in Table 2 was based on queen contiguity, wherein census tracts or block groups sharing a border, even a single point, were assigned nonnegative values in the weight matrix. As alternative specifications, we considered models with weight matrices constructed with the 4 or 8 nearest census tracts or block groups. Estimates from these models are presented in eTables 1 and 2 in the Supplement. Similar to findings reported in Table 2, estimates from models with the alternative weight matrices showed high levels of spatial autocorrelation when census tract or block group fixed effects were not included. Spatial autocorrelation continued to exist in tract-level models after the census tract fixed effects were included, but as in Table 2, there was no evidence of positive spatial autocorrelation in the census block group–level models after including census block group effects.

Given the growth in social media participation, it is possible that geographic-based social networks have become less important in recent years. Consequently, we performed alternative estimations after breaking the panels into 2 periods: one covered the 2003-2004 through 2009-2010 academic years, and the other covered the 2010-2011 through 2014-2015 academic years. Findings (eTables 3 and 4 in the Supplement) from the census tract-level models were similar to those reported in Table 2. There was no evidence of positive spatial autocorrelation in the census block group–level models in either period among estimations that included census block group fixed effects. However, the census block group–level model for the latter 5-year period provided evidence of significant spatial dependency in the model errors (λ = 0.244; 95% CI, 0.219-0.279; *P* = .002) even after the inclusion of census block group fixed effects. This could be interpreted as evidence of increased heterogeneity that transcends neighborhood, to which social media may be a contributing factor.

## Discussion

Although we did not have information on children’s actual social networks, the empirical findings reported above provide indirect evidence that social contagion is unlikely to be associated with high rates of childhood obesity in Arkansas. Of interest, there was no evidence of spatial autocorrelation in models with the smallest geographic units (ie, block groups) after geographic fixed effects were included to account for unobserved and time-invariant contextual factors. If social contagion was associated with high childhood obesity rates, we would have expected to see significant evidence of spatial autocorrelation even after inclusion of these fixed effects.

The tract-level models did continue to show positive spatial autocorrelation after the inclusion of fixed effects, but these models also showed significant spatial dependence in the error structure, which could suggest that the larger tract effects may have been too aggregate to accurately reflect key neighborhood features that were associated with obesity. The estimates of spatial autocorrelation in the tract-level models were similar to some of those estimated by Chen and Wen,^16^ who examined adult obesity rates across townships in Taiwan, a spatial scale that would be more aggregate than the tracts in our study. Similarly, Christman et al^17^ also found evidence of significant autocorrelation at the census tract level in their study of adult BMI.

Despite evidence of spatial autocorrelation at the tract level, that spatial correlation was not present at the block group level was the primary finding of this study. If social contagion were important, it would be expected in these block group–level models because geographic-based social networks are more likely to span block boundaries than tract boundaries. Thus, the findings of this study provide little evidence of an association of social contagion with the increase in childhood obesity rates in Arkansas. This is not inconsistent with a recent study by Asirvatham et al^30^ that used a natural experiment involving a court decision that resulted in the plausibly exogenous reassignment of some children in Arkansas to different schools. Although their findings provide significant evidence of social contagion, the effects that they report were small, and the results presented here are consistent with their findings. The implication for policy is that neighborhood contextual environments matter, but geographic-based social networks may not need to be the central focus of interventions.

### Limitations

There are several limitations of our study. Although geographic proximity likely plays a role in the formation of friendship networks, social networks are complex^31^ and not based solely on this, especially in the age of social media. To the extent that geographic proximity is less important to social networks, social contagion could play an important role in childhood obesity that would not be reflected in dependency across spatial units. Second, we examined autocorrelation across census-based geographic areas. Use of school-based geographic areas may be more reflective of social networks; however, school catchment areas change with time. Moreover, schools change as children progress through the public school system, with intermediate and high schools drawing children from larger geographic areas than elementary schools. Nevertheless, most children in the same block group attend the same schools, and thus we expect that the geographic controls are adequate.

## Conclusions

Social contagion and environmental factors are inherently different mechanisms that could be associated with the increase in childhood obesity. We found little evidence that geographic-based social contagion is associated with obesity rates across neighborhoods in Arkansas. Contextual factors operating at a neighborhood level were of greater importance in explaining spatial differences in obesity rates.

## Notes

Supplement. **eAppendix.** Details on the spatial autoregressive moving average (SARMA) model **eTable 1.** Census tract-level spatial panel models for alternative spatial weight matrices **eTable 2.** Census block group–level spatial panel models for alternative spatial weight matrices **eTable 3.** Census tract-level spatial panel models for alternative time periods **eTable 4.** Census block group–level spatial panel models for alternative time periods
