# Supplementary material for: Association of Neighborhood Geographic Spatial Factors With Rates of Childhood Obesity
Source: JAMA Netw Open. 2018 Aug 3;1(4):e180954. doi: 10.1001/jamanetworkopen.2018.0954 (PMC6324267; doi:10.1001/jamanetworkopen.2018.0954)
Supplement: Supplement. — eAppendix. Details on the spatial autoregressive moving average (SARMA) model eTable 1. Census tract-level spatial panel models for alternative spatial weight matrices eTable 2. Census block group–level spatial panel models for alternative spatial weight matrices eTable 3. Census tract-level spatial panel models for alternative time periods eTable 4. Census block group–level spatial panel models for alternative time periods [file jamanetwopen-1-e180954-s001.pdf]

Supplementary Online Content

Fang D, Thomsen MR, Nayga RM, Goudie A. Association of Neighborhood Geographic Spatial Factors With Rates of Childhood Obesity. *JAMA Netw Open.* 2018;1(4):e180954. doi:10.1001/jamanetworkopen.2018.0954

Supplement. eAppendix. Details on the spatial autoregressive moving average (SARMA) model

eTable 1. Census tract-level spatial panel models for alternative spatial weight matrices

eTable 2. Census block group–level spatial panel models for alternative spatial weight matrices

eTable 3. Census tract-level spatial panel models for alternative time periods

eTable 4. Census block group–level spatial panel models for alternative time periods

This supplementary material has been provided by the authors to give readers additional information about their work.

© 2018 Fang D et al. *JAMA Network Open.*

eAppendix. Details on the spatial autoregressive moving average (SARMA) model

In the equation below, $\boldsymbol{Y_{it}}$ denotes the percentage of obese children living in spatial unit *i* at time *t*, and $\boldsymbol{X_{kit}}$ is the matrix of *k* demographic variables of the school children across time. As shown in Table 1 of the article, these variables include average age, proportion by gender, race or ethnicity, and school meal status. Social contagion across nearby block groups would be captured by $\rho \boldsymbol{WY_{it}}$, where $\boldsymbol{W}$ is the spatial weight matrix. To exclude the endogenous self-influence, the diagonals of $\boldsymbol{W}$ are set to zero. $\boldsymbol{W}$ is also row-standardized to summarize a weighted-average obesity rates of all "neighbors". Therefore, the spatial autoregressive parameter, ρ, is the parameter of interest. A positive ρ indicates spillover (social contagion) in obesity across geographic space. We also assume that obesity can be influenced by the contextual environment, which we capture with census tract or census block group fixed effects denoted by $\boldsymbol{\eta_i}$. We control for time effects with $\boldsymbol{\tau_t}$ to capture changes in the environment through time. As discussed, spatial units are represented by the weight matrix $\boldsymbol{W}$ at either the census tract level or the census block group level.

$$\boldsymbol{Y_{it}} = \alpha + \boldsymbol{\tau_t} + \boldsymbol{\eta_i} + \sum_{k=1}^{K} \boldsymbol{\beta_k X_{kit}} + \rho \boldsymbol{WY_{it}} + \boldsymbol{u_{it}}$$

and

$$\boldsymbol{u_{it}} = \lambda \boldsymbol{Wu_{it}} + \boldsymbol{\mathcal{E}_{it}}$$

Because unobserved factors may not be independent of spatial locations, we assume that $\boldsymbol{u_{it}}$ follows a similar autoregressive pattern, represented by $\lambda \boldsymbol{Wu_{it}}$. This way we take care of the unobserved correlated effects that can contribute to obesity.

© 2018 Fang D et al. *JAMA Network Open.*

eTable 1. Census tract-level spatial panel models for alternative spatial weight matrices[a]

	(1) [b] Estimate (95% CI)	(2) [c] Estimate (95% CI)	(3) [b] Estimate (95% CI)	(4) [c] Estimate (95% CI)
Census tract level models (n = 8,232)				
Spatial error term (λ)	-0.077 (-0.142 to -0.012)	-0.156 (-0.314 to 0.002)	0.156 (0.082 to 0.230)	-0.197 (-0.403 to 0.008)
Spatial lag term (ρ)	0.357 (0.313 to 0.401)	0.203 (0.069 to 0.338)	0.296 (0.241 to 0.351)	0.246 (0.089 to 0.404)
African American	-0.012 (-0.017 to -0.008)	-0.060 (-0.084 to -0.036)	-0.011 (-0.016 to -0.006)	-0.060 (-0.084 to -0.036)
Hispanic	0.047 (0.037 to 0.057)	0.088 (0.053 to 0.123)	0.060 (0.049 to 0.071)	0.087 (0.053 to 0.122)
Asian	-0.269 (-0.311 to -0.227)	-0.070 (-0.151 to 0.011)	-0.277 (-0.323 to -0.232)	-0.071 (-0.152 to 0.010)
Other race	-0.389 (-0.441 to -0.337)	-0.451 (-0.511 to -0.392)	-0.363 (-0.417 to -0.308)	-0.452 (-0.511 to -0.392)
Female	-0.005 (-0.032 to 0.023)	0.005 (-0.022 to 0.032)	0.005 (-0.022 to 0.032)	0.004 (-0.023 to 0.032)
Free school meals[d]	0.104 (0.097 to 0.112)	0.026 (0.012 to 0.040)	0.113 (0.106 to 0.121)	0.026 (0.012 to 0.040)
Reduced-price meals[d]	0.107 (0.089 to 0.126)	0.004 (-0.013 to 0.021)	0.127 (0.106 to 0.147)	0.004 (-0.013 to 0.021)
Average age (years)	0.005 (0.002 to 0.007)	0.003 (0.000 to 0.006)	0.005 (0.002 to 0.008)	0.003 (0.000 to 0.006)
Year effects	Yes	Yes	Yes	Yes
Census tract effects	No	Yes	No	Yes
Weight	Four nearest neighbors		Eight nearest neighbors	

[a] Unless otherwise specified all variables are proportion of students within the census tract or census block group.
[b] Spatial autoregressive moving average (SARMA) model without geographic fixed effects.
[c] SARMA model with time and geographic fixed effects.
[d] Free and reduced-price school meals represent the proportion of lower-income children in the census tract or census block group. Children from families with incomes below 130 percent of the poverty level are eligible for free meals. Those with incomes between 130 percent and 185 percent of the poverty level are eligible for reduced-price meals.

© 2018 Fang D et al. *JAMA Network Open.*

eTable 2. Census block group–level spatial panel models for alternative spatial weight matrices[a]

	(1)[b] Estimate (95% CI)	(2)[c] Estimate (95% CI)	(3)[b] Estimate (95% CI)	(4)[c] Estimate (95% CI)
Census block-group level models (n = 25,764)				
Spatial error term (λ)	-0.327 (-0.367 to -0.286)	0.196 (0.107 to 0.284)	-0.229 (-0.286 to -0.171)	0.009 (-0.247 to 0.265)
Spatial lag term (ρ)	0.459 (0.433 to 0.485)	-0.166 (-0.267 to -0.065)	0.459 (0.427 to 0.492)	0.070 (-0.176 to 0.316)
African American	-0.007 (-0.010 to -0.004)	0.000 (-0.018 to 0.017)	-0.008 (-0.011 to -0.005)	-0.002 (-0.020 to 0.015)
Hispanic	0.027 (0.020 to 0.033)	0.086 (0.062 to 0.11)	0.029 (0.022 to 0.036)	0.085 (0.061 to 0.109)
Asian	-0.070 (-0.092 to -0.047)	0.138 (0.099 to 0.176)	-0.065 (-0.089 to -0.042)	0.137 (0.098 to 0.175)
Other race	-0.350 (-0.395 to -0.306)	-0.206 (-0.267 to -0.146)	-0.341 (-0.388 to -0.295)	-0.205 (-0.266 to -0.145)
Female	-0.057 (-0.072 to -0.042)	-0.060 (-0.076 to -0.044)	-0.058 (-0.073 to -0.043)	-0.062 (-0.078 to -0.045)
Free school meals[d]	0.086 (0.081 to 0.091)	0.024 (0.013 to 0.036)	0.091 (0.085 to 0.096)	0.023 (0.012 to 0.035)
Reduced-price meals[d]	0.080 (0.069 to 0.092)	0.009 (-0.007 to 0.025)	0.085 (0.073 to 0.097)	0.008 (-0.007 to 0.023)
Average age (years)	0.007 (0.005 to 0.009)	0.009 (0.008 to 0.011)	0.008 (0.006 to 0.009)	0.009 (0.007 to 0.011)
Year effects	Yes	Yes	Yes	Yes
Census block-group effects	No	Yes	No	Yes
Weight	Four nearest neighbors		Eight nearest neighbors	

[a] Unless otherwise specified all variables are proportion of students within the census tract or census block group.
[b] Spatial autoregressive moving average (SARMA) model without geographic fixed effects.
[c] SARMA model with time and geographic fixed effects.
[d] Free and reduced-price school meals represent the proportion of lower-income children in the census tract or census block group. Children from families with incomes below 130 percent of the poverty level are eligible for free meals. Those with incomes between 130 percent and 185 percent of the poverty level are eligible for reduced-price meals.

© 2018 Fang D et al. *JAMA Network Open.*

eTable 3. Census tract-level spatial panel models for alternative time periods[a]

	(1)[b]	(2)[c]	(3)[b]	(4)[c]
	Estimate (95% CI)	Estimate (95% CI)	Estimate (95% CI)	Estimate (95% CI)
	Census tract level models 2003/2004 through 2009/2010 Academic Years (n = 4,802)		Census tract level models 2010/2011 through 2014/2015 Academic Years (n = 4,802)	
Spatial error term (λ)	-0.251 (-0.321 to -0.181)	-0.252 (-0.404 to -0.100)	-0.251 (-0.321 to -0.181)	-0.252 (-0.404 to -0.100)
Spatial lag term (ρ)	0.511 (0.469 to 0.553)	0.271 (0.147 to 0.396)	0.511 (0.469 to 0.553)	0.271 (0.147 to 0.396)
African American	-0.011 (-0.015 to -0.007)	-0.058 (-0.081 to -0.034)	-0.011 (-0.015 to -0.007)	-0.058 (-0.081 to -0.034)
Hispanic	0.051 (0.043 to 0.060)	0.088 (0.055 to 0.122)	0.051 (0.043 to 0.060)	0.088 (0.055 to 0.122)
Asian	-0.214 (-0.253 to -0.175)	-0.062 (-0.141 to 0.018)	-0.214 (-0.253 to -0.175)	-0.062 (-0.141 to 0.018)
Other race	-0.360 (-0.409 to -0.311)	-0.447 (-0.506 to -0.388)	-0.360 (-0.409 to -0.311)	-0.447 (-0.506 to -0.388)
Female	-0.011 (-0.037 to 0.016)	0.004 (-0.023 to 0.031)	-0.011 (-0.037 to 0.016)	0.004 (-0.023 to 0.031)
Free school meals[d]	0.089 (0.082 to 0.096)	0.024 (0.011 to 0.038)	0.089 (0.082 to 0.096)	0.024 (0.011 to 0.038)
Reduced-price meals[d]	0.096 (0.080 to 0.113)	0.003 (-0.013 to 0.020)	0.096 (0.080 to 0.113)	0.003 (-0.013 to 0.020)
Average age (years)	0.003 (0.000 to 0.006)	0.003 (0.000 to 0.005)	0.003 (0.000 to 0.006)	0.003 (0.000 to 0.005)
Year effects	Yes	Yes	Yes	Yes
Census tract effects	No	Yes	No	Yes

a. Unless otherwise specified all variables are proportion of students within the census tract or census block group.
b. Spatial autoregressive moving average (SARMA) model without geographic fixed effects.
c. SARMA model with time and geographic fixed effects.
d. Free and reduced-price school meals represent the proportion of lower-income children in the census tract or block group. Children from families with incomes below 130 percent of the poverty level are eligible for free meals. Those with incomes between 130 percent and 185 percent of the poverty level are eligible for reduced-price meals.

© 2018 Fang D et al. *JAMA Network Open.*

eTable 4. Census block group–level spatial panel models for alternative time periods[a]

	(1)[b]	(2)[c]	(3)[b]	(4)[c]
	Estimate (95% CI)	Estimate (95% CI)	Estimate (95% CI)	Estimate (95% CI)
	Block-group level models 2003/2004 through 2009/2010 Academic Years (n = 15,029)		Block-group level models 2010/2011 through 2014/2015 Academic Years (n = 15,029)	
Spatial error term (λ)	-0.466 (-0.522 to -0.410)	-0.084 (-0.297 to 0.128)	-0.450 (-0.520 to -0.380)	0.244 (0.086 to 0.401)
Spatial lag term (ρ)	0.591 (0.559 to 0.623)	0.113 (-0.084 to 0.311)	0.538 (0.496 to 0.581)	-0.208 (-0.393 to -0.022)
African American	-0.009 (-0.013 to -0.006)	-0.004 (-0.028 to 0.02)	0.000 (-0.005 to 0.004)	-0.014 (-0.049 to 0.021)
Hispanic	0.026 (0.019 to 0.034)	0.097 (0.065 to 0.129)	0.040 (0.030 to 0.049)	0.043 (-0.005 to 0.091)
Asian	-0.012 (-0.038 to 0.014)	0.237 (0.19 to 0.284)	-0.118 (-0.153 to -0.083)	0.013 (-0.072 to 0.097)
Other race	-0.352 (-0.408 to -0.295)	-0.262 (-0.353 to -0.171)	-0.258 (-0.321 to -0.195)	0.002 (-0.105 to 0.108)
Female	-0.075 (-0.093 to -0.057)	-0.092 (-0.114 to -0.071)	-0.024 (-0.047 to -0.001)	-0.023 (-0.053 to 0.006)
Free school meals[d]	0.072 (0.066 to 0.078)	0.006 (-0.008 to 0.021)	0.077 (0.069 to 0.085)	0.027 (0.004 to 0.051)
Reduced-price meals[d]	0.055 (0.043 to 0.067)	0.01 (-0.005 to 0.024)	0.129 (0.106 to 0.152)	0.024 (-0.011 to 0.06)
Average age (years)	0.005 (0.003 to 0.007)	0.009 (0.007 to 0.012)	0.008 (0.006 to 0.011)	0.011 (0.007 to 0.014)
Year effects	Yes	Yes	Yes	Yes
Census block-group effects	No	Yes	No	Yes

a. Unless otherwise specified all variables are proportion of students within the census tract or census block group.
b. Spatial autoregressive moving average (SARMA) model without geographic fixed effects.
c. SARMA model with time and geographic fixed effects.
d. Free and reduced-price school meals represent the proportion of lower-income children in the census tract or census block group. Children from families with incomes below 130 percent of the poverty level are eligible for free meals. Those with incomes between 130 percent and 185 percent of the poverty level are eligible for reduced-price meals.

© 2018 Fang D et al. *JAMA Network Open.*
